# Establishing single cell RNA transcriptomics: a brief guide

**DOI:** 10.1186/s12983-025-00579-x

**Published:** 2025-09-02

**Authors:** Alison G. Cole

**Affiliations:** Department of Neurosciences and Developmental Biology, University of Vienna, Vienna, Austria

**Keywords:** Cell dissociations, Cell-type inventories, Single cell RNA sequencing

## Abstract

Single cell RNA sequencing is a tool for evaluating the specific transcriptome usage of different cell types within an organism. By tagging mRNA molecules from single cells or nuclei, a non-biased assay of the active transcriptome is captured. The method relies on high-quality cell suspensions, which can be challenging to obtain from whole organisms. While the costs per cell are rapidly falling as this technology matures, there is still a requirement for a non-trivial economic investment. Data analyses pipelines are also rapidly maturing, yet gold standards for data integration methods and trajectory inference are still lacking. Here, I review the standard procedures for generating these data from emerging models and highlight prerequisites to consider during project design, including the choice between cells and nuclei, fresh or fixed material, target capture numbers and methods, sequencing depth, and finally expected analysis outcomes.

## Background

Over the past decade, advances in microfluidics and low-input RNA sequencing have opened the floodgates for sequencing transcriptomes from single cells [1] or single nuclei [2]. In this review the term ‘single cell’ refers to data generated from either intact cells or nuclei unless otherwise specified. In 2015, the field exploded when these low-input RNA processing techniques were combined with microfluidics, driving fluids through small channels to combine molecular biology reagents, a single cell, and a primer-delivery bead, all encapsulated within a single drop embedded in an oil emulsion, thousands of times-over [3, 4]. Low-input RNA sequencing methods applied to whole organism biology have led to the generation of transcriptomic profiles from blastomere-derived cell colonies [5, 6], time courses of single- embryos [7], profiling of entire organisms [8], and finally single cells from time-courses of entire embryos [9, 10]. Analysis methods for these new data then exploded, with relatively standard pipelines now available in several programming languages (e.g.: R: Seurat [11–15]; Python: Scanpy [16]). This review will summarize the key steps necessary for the application of single-cell RNA sequencing to novel and emerging non-model systems, highlighting common pitfalls to consider, and providing considerations for overall project design.

## Principle and applications

**Fig. 1:**
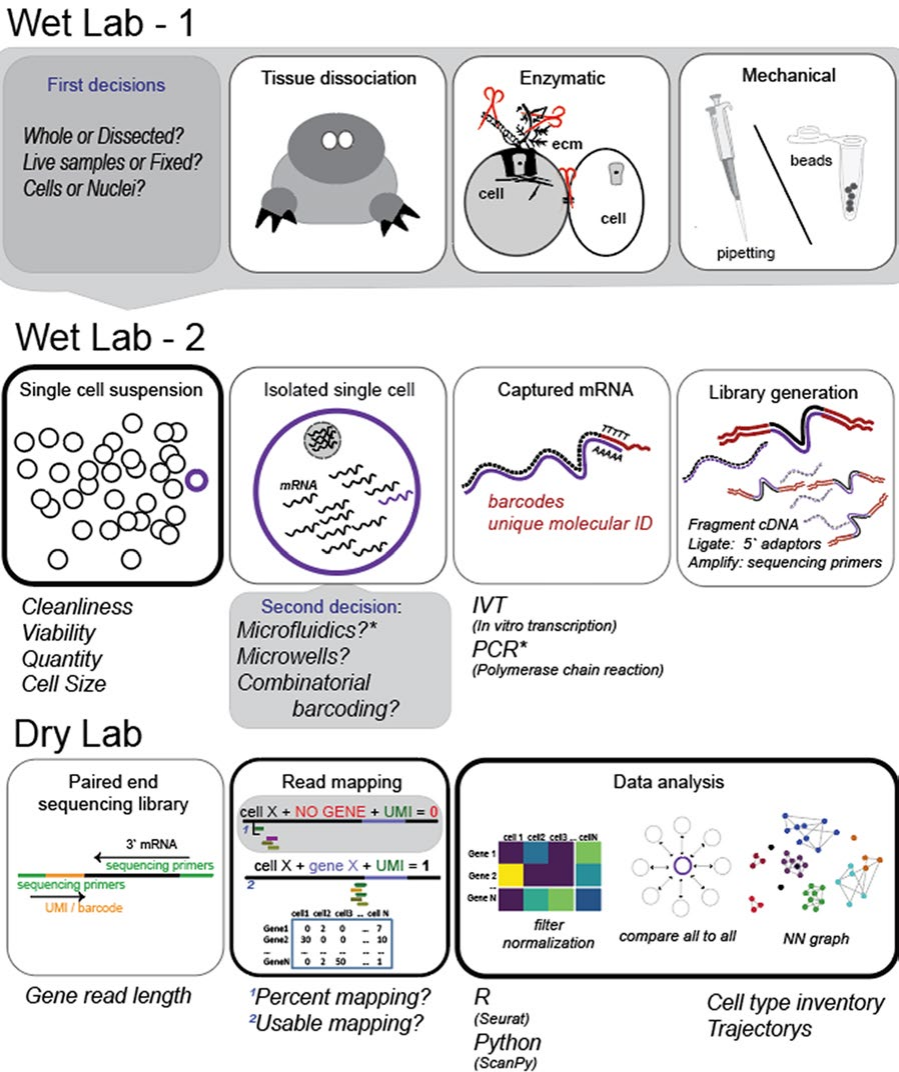
Overview of a single cell/nucleus transcriptomics sequencing experiment. Wet lab part 1 involves making decisions regarding sample type and generating the samples. Wet lab part 2 requires selecting the cell capture method and generating sequencing libraries. *Indicates most commonly used methods. The Dry lab then demultiplexes the sequencing data to generate an expression matrix that is used for downstream analysis

Single cell transcriptomic profiling involves three basic steps, each one with its own challenges and limitations (Fig. 1). The first step involves preparing the samples for data acquisition. Ultimately, this means converting the tissue of interest into a quality single cell or nuclei suspension. The second step is to isolate single cells/nuclei of interest, tag their mRNA molecules with a poly-A oligo, and generate a three-prime biased sequencing library which is sequenced with a paired-end sequencing strategy. Finally, data analysis is performed. This involves mapping the reads to an adequate reference to generate a count matrix, followed by downstream bioinformatic analyses of the expression profiles.

**Fig. 2:**
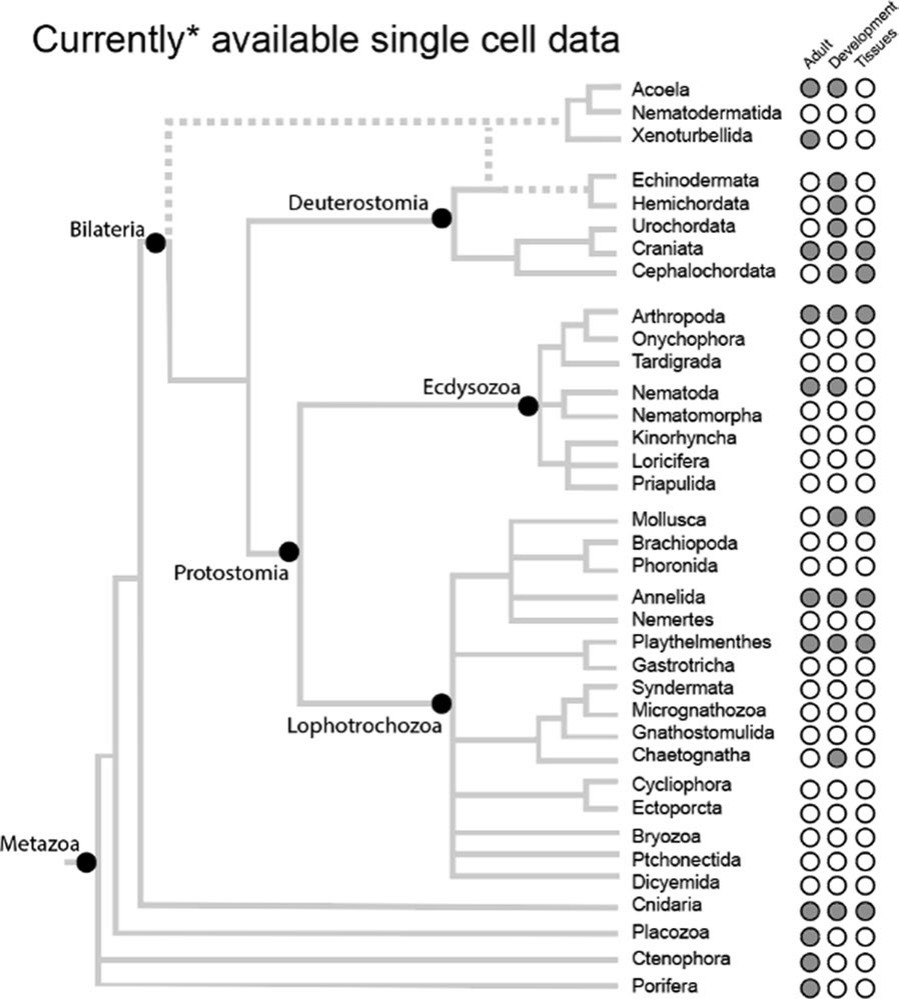
Distribution of currently available single cell datasets as of data of submission of the review. Green dots indicate available data for at least one taxon in the depicted lineage. References are available in the main text

Once single cell transcriptomic data are generated, these are initially used to generate an inventory of transcriptomic states present in the sample. This can be as comprehensive as providing a catalog of cell types for an entire organism, or as focused as searching for a specific cell type, for example multipotent stem cells [17–21]. Amongst the invertebrates, single cell inventories are now available for members of the more basally branching clades Porifera [22, 23] and Ctenophora [22], the bilaterian sister groups Placozoa [24] and Cnidaria [19, 21, 25–30], as well as bilaterians belonging to the Acoela [18, 31], Lophotrochozoa [17, 20, 32–37], Ecdysozoa [38–41], and Deuterostomia [42–49], and other enigmatic taxa (Xenacoelomorpha [50]; Chaetognatha [51]) (Fig. 2).

## Establishing the technique

### Considerations before starting

There are two principal requirements necessary prior to embarking on a single cell sequencing project. The sequencing data that you receive from these experiments can only be interpreted if the sequences can be assigned to gene models with functional annotations and putative orthologies. When available, mapping sequencing reads to a genome with complete gene annotations gives the most flexibility. If such a genomic resource is unavailable, it will be necessary to invest in generating at least a transcriptome assembly [52, 53]. In parallel, generating the sequencing data requires a protocol for cell or nuclei suspensions from your tissue of interest, either from a dissected tissue or a whole small animal. This is a non-trivial hurdle for many non-model organisms and could require several months of experimental wet-lab trials to develop a working protocol. The decision to sequence single cells or single nuclei depends also on the intended use of the data. For many applications entire cell capture is ideal, as the number of mRNAs within the cytoplasm is greater than that of the nucleus [2, 54]. Cells that are particularly difficult to isolate, for example neurons, can benefit from nuclear isolation. Here, the cytoplasmic component of the cells is discarded, and the expression profiles are restricted to genes that are being actively transcribed. This could be detrimental for studies that rely on cytoplasmic distribution of mRNA, but in general single nuclei give data that are readily comparable to single cell counterparts [2, 55], although there are some use-cases where this is not so [56], and some cell types show different distributions in nuclear versus intact cellular samples [57, 58]. Single nuclei sequencing is also compatible with multiome studies, combining transcriptomes with open chromatin (ATAC-seq). Once these two pre-requisites are met it is relatively straight-forward to generate these data, and the costs involved for both producing, and sequencing, a single cell transcriptome library are constantly improving as new solutions enter the market.

The choice of starting material is of course directly related to the biological question being interrogated with the data. Generating a comprehensive inventory of cell types for an organism requires dissociation of all its tissues. To achieve this, we often prepare multiple samples from separate dissections. This strategy allows for limited spatial information to be retained and enables the use of customized dissociation protocols tailored to the varying characteristics of different tissues [21, 28, 59]. This is the approach taken by the human cell atlas (https://data.humancellatlas.org/), where dissociation of an intact individual is not possible. Thus if your primary research interest is for example a specific cell type that can produce silk within the silkworm, then it makes sense to reduce the complexity of the data by first performing a clean dissection of the tissue and discarding the rest [60]. Some tissues are more challenging to achieve clean suspensions due to extensive extracellular components, or the presence of very fragile cells. In this case fluorescence-activated cell sorting (FACS) with commercially available live/dead stains can be used to eliminate debris from cell suspensions, but runs the risk of introducing artifacts related to cell stress during the sorting process [58, 61], or losing specific cell types that are more fragile than others (for example larval blood fluke ciliary plate cells [34]). Even the dissociation introduces transcriptomic responses in the cell populations and so performing digestions on ice can help mediate these transcriptional responses. However, this approach may slow digestion times because most commercially available enzymes are optimized for activity at 37C. Recently, fixation-based methods have been applied to relieve some of these issues by essentially stopping the transcriptomic response. Using fixed material for FACS is therefore preferable, whether it be part of the dissociation process itself (methanol maceration optimized for single cell sequencing [ACME] [62]), or reversable dithio-bis(succinimidyl propionate) (DSP) fixation immediately following the cell dissociation [63]. In addition to removing debris from the suspensions, FACS is invaluable for specific cell enrichment, allowing for collection of fluorophore-expressing cell lines (for example cnidarian neurons [27]) or sorting according to antibody labeling for any available antibody (for example, lizard neural crest cells [64]). Often the best approach will be dictated by the source material and the desired information to be obtained.

### Reagents and resources

Current commercially available solutions for cell capture and library generation vary with respect to how cells are collected and thus have different minimal input requirements (Table 1). For example, 10× genomics offers a droplet microfluidics solution with the flexibility to capture as few as 500, or as many as 20,000 cells with their latest GEM-X v4 assay. Similarly, Illumina now offers a single cell droplet capture solution that is vortex-based ([65], commercialized by Fluent Biosciences, purchased by illumina 2024) and can process a wide range of input without the restriction of a microfluidics platform and thus eliminating any size-related restrictions related to channel width or microwell size. Other solutions involve sorting cells into microwells (BD Rhapsody, Singleron) with a much larger maximal size capacity than microfluidics approaches. Plate based combinatorial barcoding solutions [66], such as that offered by Scale BioScience and Parse BioScience, return over 100,000 cells and as such have the lowest cost/cell. However, this technology requires as input a minimum one million cells and so may be unsuitable for smaller projects. Most often costs are quoted as cents/cell but bear in mind that the more cells that are captured in a single run, the lower these costs will be. Smaller targeted projects will thus have a greater per cell cost. This also fails to consider the sequencing costs, which will require about 20,000 paired-end reads per cell and thus scales sharply as the number of cells captured increases.

**Table 1:** Features of commercial capture platforms

Commercial solution	Capture platform	Hardware needed	Throughput (Cells/Run)	Capture efficiency (%)	Max cell size	In assay sample multiplexing	Samples/run	Nuclei capture	Live cell capture	Fixed cell support
10× Genomics Chromium	Microfluidic oil partitioning	Yes	500–20,000	70–95	30 µm	4 Samples	1–8	Yes	Yes	Yes
BD Rhapsody	Microwell partitioning	Yes	100–20,000	50–80	30 µm	12 (Mouse/Human only)	8	Yes	Yes	Yes
Singleron SCOPE-seq	Microwell partitioning	No/Yes	500–30,000	70–90	< 100 uM	Up to 16 samples	1–4	Yes	Yes	Yes
Parse Evercode	Multiwell-plate	No	1000–1M	> 90	–	Up to 384 samples	1–96	Yes	No	Yes
Biosciences Quantum Scale	Multiwell-plate	No	84K–4M	> 85	–	Up to 96 samples	1–96	Yes	No	Yes
Fluent/PIPseq (Illumina)	Vortex-based oil partitioning	No	1000–1M	> 85	–	No	1	No	Yes	Yes

Platforms differ in the number of cells they can process, whether they are compatible with fixation, and how many different samples can be processed into a single sequencing library (multiplexing)

### Cost assessment

Cost categories to be considered when planning a single cell sequencing experiment include sample preparation (Fig. 1: Wet Lab 1—tissue dissociation, cell sorting, viability assays), library preparation (Fig. 1: Wet Lab 2—reagents and kits for chosen platform), and sequencing costs (Fig. 1: Dry Lab—driven by read depth per number of cells). Computational resources and data storage requirements grow substantially with large datasets and may incur additional costs. In small-scale projects, library preparation and per-sample sequencing often dominate the budget, making platform choice and cell number especially critical. In contrast, for large-scale projects sequencing costs tend to become the main budget driver. This is particularly true when profiling many samples or aiming for high-resolution coverage. Strategic planning, such as optimizing cell numbers, multiplexing samples, or adjusting sequencing depth, can help balance cost and data quality across different project sizes.

### Performing the proof of principle experiment

#### WET LAB 1: Generating the input material: single cell/nuclei suspension

Optimizing single-cell dissociation and nuclei isolation is critical for ensuring high-quality, viable material. This is the first and often the most challenging step for any tissue or organism of interest. There are many options for outsourcing the generation of single cell sequencing libraries, but generally these services will require delivery of a cell suspension with targeted concentrations at a minimum volume. Live cell dissociations typically include a combination of enzymatic digestion of extracellular components and mechanical stresses to essentially pull the cells apart. This needs to be done rapidly so that transcriptomic response to the dissociation is minimized. Most often mechanical stress is applied by moderate pipetting of the suspension; however larger tissue pieces may require other methods to sufficiently break down the tissue. If the starting tissue is abundant, cutting into small fragments (1–3 mm^3^) will enhance enzymatic penetration. Mechanical stimuli range from oscillations on a shaker, frequent pipetting, or the use of mortar and pestle, beads, or other commercial solutions (i.e. gentleMACS: Miltenyi Biotec; TissueLyser: Qiagen). For small marine larvae removal of cations that mediate cell–cell junctions can be sufficient to obtain enzyme-free single cell solutions (echinoderm larvae: [47, 48]). Samples rich in extracellular matrix can benefit from collagenase-based digestions, but this may elicit a transcriptomic response [67], while protease-based digestions aid in breaking cell–cell interactions. These two enzyme types are often used in combination, and optimized mixes have been developed with this purpose in mind (i.e. Liberase by Roche). Enzymatic digestion must be carefully optimized in terms of enzyme concentration, incubation time, and temperature to maximize cell yield while minimizing stress and preserving RNA integrity. Preliminary experiments should start with a spectrum of enzyme concentrations over a series of time points and assess the cell viability and single-cell-ness of the solution at the end. Clumpiness of a cell suspension can be monitored by placing a few microliters of the suspension on a slide and viewing it with a compound microscope. This is also useful for detecting when the conditions are too harsh: suspensions should be free of nuclei or other cellular debris. It is important to keep in mind that small cell multiplets will be captured and sequenced together, as will cytoplasmic blebs or other debris. While trypan-blue exclusion provides a preliminary estimate of cell viability, fluorescence-based assays, typically using fluorescein or its derivatives (live) and propidium iodide (dead), offer a more accurate assessment of viability and allow precise cell quantification prior to capture. Regular measuring of viability estimates under the microscope as the dissociation protocol is optimized for a new system will give an idea of the maximum viability for that tissue/organism that can be expected when preparing the final sample for sequencing. As a general guideline when troubleshooting a new dissociation protocol, low yields most often indicate insufficient dissociation. Dissociation protocols often include cell strainers to remove large undigested tissue pieces; to avoid clogging these filters, visible tissue clumps should be removed prior to filtering. Low cell viability indicates the treatment has been overly harsh; in this case check for cellular debris and free nuclei as indications of over-digestion. The goal is to achieve the highest viability (> 80% is ideal, but sometimes this just isn’t achievable) in the shortest timeframe (less than 30 min is ideal), with the greatest percentage of single cells. When whole-cell dissociation is not feasible, or nuclei are desired for multiomic protocols, tissues are usually first homogenized mechanically in a hypotonic or detergent-based lysis buffer that disrupts the plasma membrane while preserving nuclear integrity. The lysis conditions must be carefully titrated to prevent nuclear rupture or clumping. Following lysis, nuclei can be filtered through a cell strainer and washed in BSA-containing buffer to remove cytoplasmic debris and reduce background RNA. Buffers used should be RNase-free and samples must be kept on ice whenever possible to slow transcriptomic responses to the dissociation process.

#### WET LAB 2: Generating the raw data: cell capture, barcoding, sequencing

Once the cell/nuclei suspension is ready, an isolation method is applied. This can be as ‘simple’ as manually collecting individual cells into separate tubes [40, 68] or multi-well plates [66], or as ‘complex’ as sorting into plates with the assistance of a pipetting robot or FACS machine [69], or running the samples through a micro-fluidic chip for capture into droplets within an oil-emulsion [3, 4]. Alternative techniques involve formation of these droplets in the absence of microfluidic channels, using mechanical agitation to randomly capture cells, barcoded beads, and reagents for the retro-transcription [65]. Whatever the method, the isolated samples are then barcoded for both individual cell/nuclei and molecules. This is achieved by a retro-transcription reaction that incorporates a single barcode sequence and a random nucleotide sequence into an oligo with a sequencing adaptor and a poly-A tail for binding to the mRNA of the sample. At this point the cDNA is amplified, which is predominantly done with PCR, but some protocols will use in vitro transcription to amplify the RNA, giving a more linear amplification of the starting material [5, 69–71]. One can also avoid isolating single cells by using successive rounds of barcoding of small batches of cells within multi-well plates with cell mixing occurring between each barcoding round. Costs associated with the cell capture vary widely depending upon whether specialized equipment is necessary or not (Table 1). Once the samples are barcoded, large-scale multiplexing occurs during sequencing library preparation. The cDNA is fragmented, sequencing primers are ligated, and the three-prime fragments of interest are selectively amplified by PCR. The resultant library is then sequenced with a paired-end strategy, wherein the first read will recover the barcoding information but then fall into the poly-adenylated sequence. The second read will recover a legible gene sequence from the three-prime untranslated region and the first exon of the transcript. To date this has been achieved on the illumina sequencing platform, however other sequencing options are now on the horizon (i.e. Ultima Genomics [72], Avidity [73]), including long-read sequencing options to detect single cell isoforms (i.e. PacBio [74], Oxford Nanopore [75]; reviewed in [76]).

#### Accessing the raw data: generating the data matrix

The second hurdle is to have an adequate mapping reference onto which the sequencing data can be aligned. This will either be a genome with genes predicted, or a transcriptome. In a perfect world, 100% of the sequencing reads will align with confidence to the reference genome or transcriptome and will be assigned to a single gene. In practice, these values are much lower, especially for emerging models with fewer resources available for perfecting genome assemblies and associated gene sets. Because the technique relies largely on the poly-adenylated tail of the messenger RNA, captured sequences are heavily biased towards the 3-prime end of the genes, often falling within the untranslated regions (UTR) of the genes. Thus, to accurately map the sequences to a coding gene, the three prime ends of the gene models require some attention. Ideally, one has a quality genome assembly with well annotated genes [52]. More realistically for emerging model systems, long-read transcriptome data is invaluable in this regard in that these data will provide also the often poorly annotated three-prime untranslated regions of the genes [53]. In the absence of full-length transcriptome data but having a genomic sequence at hand, it is possible to improve single cell mapping simply by extending the gene along the genome in the three-prime direction, taking care to avoid overlapping into the next gene [21, 27]. Tools are also being developed that will help improve genome-based mapping by incorporating read alignments to recover unassigned peaks and use these to extend gene models [77, 78]. However, a well-annotated genome is not strictly required, as reads can also be mapped directly to a compiled transcriptome. The single cell data itself can also be used to improve transcriptome-based mapping tools by providing strand information and anchoring and extending the models into the three-prime direction [7]. Having a well assembled transcriptome is only the first step, as the single cell transcriptome profiles receive meaningful interpretation from analysis of the collection of functional annotations of the genes that comprise the profiles. Thus, having gene orthology information available associated with the mapping tool is also necessary. Furthermore, associating single cell gene expression data with cross-species gene orthology information allows for the identification of similar cell types across species (for example: [59, 79]). This is a branch of the field that is still in its infancy and so is not further discussed here but the reader is referred to the following resources for more information [80–83].

Commercial platforms offer pipelines for sequencing alignment and subsequent demultiplexing. Once the sequences have been aligned to the reference, they are binned by gene, sample, and unique molecular identifier (UMI) so that reads are only counted if they fulfill three criteria: unique mapping to a single reference gene, from the same sample, and with an identical UMI = 1 read. Genes that are represented by multiple molecules have multiple UMIs binned to the same cell and gene, therefore increasing the count number in the resulting matrix. Duplicate UMIs that are the result of PCR amplification and thereby eliminated, and the degree of UMI collapse can be used as an estimate of sequencing saturation. The deeper a sample is sequenced, the less likely it becomes to recover a unique read within the library. The amount of sequencing required for a single sequencing library will depend upon how many cells/nuclei were captured, and how many cycles of PCR amplification were used to incorporate the sequencing primer sites. Over-amplification can reduce the sensitivity of the assay, reducing the ability to detect lowly expressed transcripts. As a ball-park estimation, 10× genomics for example currently recommends sequencing a minimum of 20,000 read pairs per cell. Ultimately, if the sequencing saturation estimate falls below 80% then your sequencing libraries may benefit from additional rounds of sequencing. This is important to remember when designing the experiments, as more cells captured lowers the cost per/cell for generation of the sequencing library, but the sequencing investment will be higher to reach sequencing saturation per cell.

#### DRY LAB: Processing the data: basic workflow

The third hurdle is the data analysis itself. All commercial kits will provide a bioinformatics workflow to generate a count matrix from the sequencing data, and often also provide a standard clustering output. For further data exploration there are two principal platforms available for downstream data analysis, whether one is more familiar with R or Python coding languages. There are very well documented packages available for both environments that essentially perform the same basic workflow (i.e. Python: Scanpy [https://scanpy.readthedocs.io/en/stable/]; R: Seurat [https://satijalab.org/seurat/]). A community-driven manual documenting best practices is available here: https://www.sc-best-practices.org/preamble.html [84]. Count matrices are first filtered for minimal information (numbers of molecules and genes mapped in each cell), and often also for putative multiplets (more than one cell tagged with the same barcode). In most current pipelines, cells that pass these filtering steps are first normalized for read count by dividing by the total reads in a cell, multiplying by a scale factor, and taking the log transformation after a pseudo-count addition. Alternatives are available, and interested readers can see [85, 86] for review and discussion of single cell data normalization. A large proportion of the resultant data matrix will show little variation across all samples, and so the most highly variable genes are identified and used as input for a first round of dimensional reduction; the standard practice is to sample the top 2000 variable genes. More heterogeneous datasets could benefit from including a larger initial gene set. While several dimensional reduction options are available (reviewed in [87]), the most frequently used algorithm is the principal component analysis. The resultant reduced dimensions are again filtered to select those that capture the most variability in the dataset. This of course will be influenced by the initial gene set. The selected dimensions are then used to construct neighbourhood graphs that depict the similarity between samples. These graphs are then used for clustering or further reductions to generate cell plots (tSNE/UMAP/force-atlas/Picasso) that are useful for intuitive visualization of the underlying data.

#### Analysing the data: generating the cell state inventory

Recently there has been heavy criticism directed towards the value of these cell plots [88], most importantly highlighting dangers of mis-interpretation of the underlying data due to the partially random configurations generated by these reduction methods. This is often hidden from the wet-lab biologist that is new to the field, as all the well-documented tools include a fixed starting variable so that the output is repeatable each time the algorithm is applied rather than having a random start site that introduces variability. However, there is only so much multi-dimensional information that can be conveyed in two- or sometimes three-dimensions. Thus, overlapping points on these visual interfaces may not always accurately reflect the underlying transcriptomic profiles. For this reason, it is important to explore the dataset in multiple ways and seek coherence across analyses and with the biological information that you have from the lab. The most frequent question newcomers have regarding these types of analyses involves clustering: what degree of clustering is enough? The answer depends largely on what the goals are. For the non-model system, the primary goal for applying single cell sequencing technologies is to generate an inventory of transcriptomic states and relate these to cell types within the organism. For this task, iterative clustering is a valuable strategy. In this case, the dataset can be first partitioned into low-resolution clusters of high similarity. This will largely correspond to the principal cell types in the dataset, separating epithelia, muscle, neurons, etc. From this clustering, one can generate lists of genes that are differentially expressed across populations and identify marker genes that are highly specific for each cluster. Iterative clustering then involves taking a subset of the data, including only cells that are very similar transcriptomically, and then selecting the variable genes from within that population. This approach allows for refinement of similar cell states that were hidden with the initial gene selection from the entire heterogeneous dataset. This allows for separation of cell states often hidden due to convergent gene expression. For example with this approach we were able to identify two distinct fast-retracting muscle cell types from a sea anemone that share a contractile apparatus presumably optimized for fast retraction [89]. An alternative approach is to use a larger set of input genes and resultant principal components to attempt to capture all variation within the dataset and start with over-clustering the dataset [17, 19]. Clusters can then be evaluated for a minimum number of cluster-specific genes, and iteratively merged with their nearest neighbour when the separation is not supported by unique gene sets [23]. This approach risks increasing noise related to technical differences when multiple samples are included in the experiment.

#### Additional considerations: multiple samples

When multiple samples are collected, either as technical or biological replicates, or as part of a more complex experimental design involving different tissues or developmental stages, it is often necessary and beneficial to merge them into a single dataset. Whether or not computational integration is necessary to identify transcriptomic similarity across samples can be assessed using the dimensional reduction cell plots; if all cells separate according to the sample of origin, then additional processing is necessary. In my experience, comparing relative expression values across samples often ameliorates any differences stemming from technical differences between samples while maintaining biological signal [21, 28]. This likely reflects the fact that cells interpret relative rather than absolute values of gene expression (for example epithelia differentiation in *Hydra* [90]). This approach is not always sufficient and so integrating single-cell transcriptomic datasets across batches, conditions, or technologies is essential to overcome batch effects and enable comparative or joint analyses. Common computational approaches include mutual nearest neighbors (MNN) correction [91], canonical correlation analysis (CCA) as implemented in Seurat [13], and Harmony, which uses iterative clustering and embedding alignment [92]. These tools allow researchers to combine data across samples while preserving biological variability, and a benchmarking study is available here, while the issues are reviewed in [93, 94].

#### Next steps: Cluster validation and further data exploration

Finally, where in situ hybridization is possible it can be informative to select specific cluster markers from the analyzed dataset for spatial detection. For well-studied models there will be expression profiles available that can be used to guide cluster identification for previously studied cell types, however the power of the single cell transcriptomic approach is the identification of novel cell states within the dataset. These previously unknown cell types can benefit from ISH analysis to place these cells in the context of the intact organism. For example the distribution of glial cells within the cephalopod brain was uncovered through a combination of comparative single cell transcriptomics and in situ hybridization [95]. Application to developmental processes such as reconstruction of cell specification pathways is another use of single cell RNA sequencing data. By modeling continuous transcriptional changes, scRNA-seq provides insight into the regulatory mechanisms driving cell fate decisions. Computational tools infer the ordering of cells along developmental paths (a.k.a. pseudotime) and can reveal lineage relationships and branching points. These approaches have been used to map differentiation hierarchies for example in the ventral nerve cord of the *Drosophila* [96] and early embryonic development in tunicates [44]. No single method has emerged that out performs the others [97], and additional approaches that integrate artificial intelligence are emerging (for example: [98, 99]).

#### Troubleshooting

There are a number of steps that require optimization in order to achieve a quality cell suspension and subsequent access to the expression data.

*Problem*: Incomplete dissociations—cell clumps remain within suspensions.

*Solutions*: Monitor dissociations by checking 2–5 uL of the suspension with a compound microscope. Consider adding filtration steps to remove large indissociable tissue pieces. Start with smaller tissue pieces.

*Problem*: Poor cell recovery—limited cells in suspension after washing

*Solutions*: check centrifugation settings; explore density gradients; consider FACS clean up

*Problem*: Poor cell viability

*Solutions*: Minimize enzyme incubations; increase washes; consider fixation approaches

*Problem*: Poor mapping

*Solutions*: extend gene models in the three prime direction (genome), re-assemble transcriptome and include single cell reads. Consider investing in long-read, full-length transcript sequencing from a bulk RNA sample of a similar tissue to improve the quality and completeness of the transcriptome.

## Conclusions

The establishment of single-cell RNA sequencing in emerging model organisms represents a transformative step in understanding cellular diversity and gene function across a wide range of biological systems. Proof-of-principle experiments demonstrate the feasibility of generating high-quality transcriptomic data. New users should carefully consider their experimental goals when evaluating the commercial platforms available. Compatibility of scRNA-seq methods with fixed cells is alleviating many of the technical hurdles associated with cell collections and building more complex experimental designs. Compatibility with multimodal omics (for example scATAC-seq) may influence the choice of optimizing nuclei dissociations over intact cells. It is advisable to begin with a pilot experiment, including shallow sequencing, to confirm that all steps are functioning properly before committing to a larger, high-depth sequencing effort. As more single cell transcriptomic data become available, this will enable deeper insights into cell differentiation pathways, lineage relationships, and the evolution of cell types. The integration of scRNA-seq datasets across species and conditions holds the promise of creating comprehensive biodiversity cell atlases, offering unprecedented opportunities to explore conserved and divergent molecular mechanisms. As sequencing technologies become more refined the ability to extend these data to full transcript sequencing from single cells will undoubtedly open the horizons for examining isoform usage across different cell types and cell states. By continuing to innovate and adapt scRNA-seq methodologies, these advancements will unlock new frontiers in developmental biology, regenerative medicine, and evolutionary studies.

## Data Availability

No new materials were generated for this work.

## Abbreviations

FACS: Fluorescence-activated cell sorting
scATAC-seq: Single cell Assay for Transposase-Accessible Chromatin using sequencing
scRNA-seq: Single (cell or nuclei) RNA sequencing
UMI: Unique molecular identifier
UTR: Untranslated region
